# Cross-Kingdom Regulation of Plant-Derived miRNAs in Modulating Insect Development

**DOI:** 10.3390/ijms24097978

**Published:** 2023-04-28

**Authors:** Xuepeng Chi, Zhe Wang, Ying Wang, Zhenguo Liu, Hongfang Wang, Baohua Xu

**Affiliations:** 1 College of Animal Science and Veterinary Medicine, Shandong Agricultural University, Tai’an 271002, China; xixuepeng@sdau.edu.cn (X.C.); 2020110449@sdau.edu.cn (Z.W.); wangying@sdau.edu.cn (Y.W.); zgliu@sdau.edu.cn (Z.L.); whf@sdau.edu.cn (H.W.); 2 Key Laboratory of Efficient Utilization of Non-Grain Feed Resources (Co-Construction by Ministry and Province), Ministry of Agriculture and Rural Affairs, College of Animal Science and Technology, Shandong Agricultural University, Tai’an 271018, China

**Keywords:** miRNAs, cross-kingdom regulation, plants, insect development

## Abstract

MicroRNAs (miRNAs), a class of non-coding small RNAs, are crucial regulatory factors in plants and animals at the post-transcriptional level. These tiny molecules suppress gene expression by complementary oligonucleotide binding to sites in the target messenger. Recently, the discovery of plant-derived miRNAs with cross-kingdom abilities to regulate gene expression in insects has promoted exciting discussion, although some controversies exist regarding the modulation of insect development by plant-derived miRNAs. Here, we review current knowledge about the mechanisms of miRNA biogenesis, the roles of miRNAs in coevolution between insects and plants, the regulation of insect development by plant-derived miRNAs, the cross-kingdom transport mechanisms of plant-derived miRNAs, and cross-kingdom regulation. In addition, the controversy regarding the modulation of insect development by plant-derived miRNAs also was discussed. Our review provides new insights for understanding complex plant–insect interactions and discovering new strategies for pest management and even crop genetic improvement.

## 1. Introduction

MicroRNAs (miRNAs) are a class of endogenous, highly conserved, single-stranded non-coding small RNAs (sRNAs) ranging in size from 18 to 23 nucleotides. They act as master modulators of target gene expression at the post-transcriptional level by binding to the 3′-untranslated region (3′-UTR) or open reading frame (ORF) [1]. MiRNAs have been demonstrated to play crucial roles in various biological processes, including cell growth and differentiation, cell proliferation, immune response, and cell apoptosis [2,3,4]. Since the first identified miRNA, lin-4, was discovered in *Caenorhabditis elegans* (Maupas, 1899) (Nematoda: Rhabditida) in 1993 [5,6], tens of thousands of miRNAs have been identified in mammals, plants, and microorganisms [3,7]. To date, the most commonly used miRNA database, miRBase version 22.1 (https://www.mirbase.org), has registered 38,589 miRNAs from 271 organisms [8].

Insects are the most abundant group of animals on earth, and miRNAs play an important role during the growth and development of insects. Studies have shown that some miRNAs play important roles in insect metamorphosis and oogenesis by regulating the hormone synthesis level or gene expression level in Kr-h1 and Notch signaling pathways [9]. MiRNAs play major and diverse roles in the biology of *Drosophila melanogaster*, ranging from germline development, maternal to zygotic transition, tissue growth, and physiological activity of the central nervous system [10]. There are significant differences in the miRNA and transcriptional profiles of diploid females relative to haploid drone males, and between reproductively distinct females, indicating that miRNA plays a crucial role in caste determination in the honeybee [11]. In addition, miRNAs are potential targets for insect pest control applications [12]. Studies on insect miRNAs have demonstrated their important roles in insect development, and there is a wide range of fields to explore in reproductive manipulation, caste differentiation, and so on.

Recently, the discovery of plant-derived miRNAs showing cross-kingdom abilities to regulate gene expression in mammals, insects, and even viruses has prompted exciting discussion [13,14,15]. The digestive system of animals can absorb plant miRNAs and release them into the circulatory system, which then delivers them to target cells to regulate the functions of recipient cells [16]. In 2012, Zhang et al. [17] reported that plant-derived miRNAs were present in the sera and tissues of various animals and that these exogenous miRNAs were primarily acquired through food intake. They found miR168a from rice could bind to the human/mouse low-density lipoprotein receptor adapter protein 1 (LDLRAP1) mRNA, inhibit LDLRAP1 expression in the liver, and consequently decrease LDL removal from mouse plasma. In 2014, miR172 from *Brassica oleracea* was also detected in the blood, spleen, liver, and kidney in mice [13]. The abundance of plant miR159 in human serum is negatively correlated with the incidence and progression of breast cancer, and oral miR159 mimics significantly inhibit the growth of mouse xenograft breast tumors [18]. Plant miR5338 was also enriched in the posterior lobes of the prostate gland of rats fed with rape bee pollen, suggesting that plant-derived miR5338 has potential utility in the treatment of rat benign prostatic hyperplasia through inhibiting Mfn1 in the prostate [19]. These findings indicate that plant-derived miRNAs can be absorbed into animals through the gastrointestinal tract and remain stable and selectively functional in target organs, target tissues, or target cells, with subsequent physiological effects. Although the findings of cross-kingdom regulation by plant-derived miRNAs are exciting, several groups of researchers have questioned these discoveries due to a lack of reproducibility [20]. Therefore, these findings must be examined dialectically.

Mutualism is a crucial outcome of the interactions between multiple species that provide the energy, nutrients, and services for ecosystems to function and persist. Over the course of their long-term evolution, plants have evolved strategies to deter herbivores whilst attracting beneficial insects. The interactions between plants and herbivorous insects are close and complex. Flowering plants depend on insects for pollination, while insects have been feeding on plants for 400 million years [21]. Studies have found that after feeding on melon phloem sap, plant-derived miRNAs can be detected in *Aphis gossypii* tissues [22]. This finding indicates that plant-derived miRNAs can enter insects and exhibit the potential to exert regulatory effects. In addition, the coevolution of plants and insects may depend on cross-kingdom regulation by plant-derived miRNAs. However, knowledge of the involvement of miRNAs in these reciprocal interactions is in its infancy [23]. The influence of miRNAs on coevolution between plants and insects is also unclear.

Although the understanding of cross-kingdom regulation by plant-derived miRNAs in insects is advancing rapidly, controversies in the field should not be ignored. The objectives of the review are to analyze current evidence regarding cross-kingdom regulation between plants and insects to enhance the understanding of the novel abilities of miRNAs and enrich the theory of insect and plant coevolution. In Table 1, the recent studies regarding cross-kingdom regulation by plant-derived miRNAs in animal systems, especially insects, are shown. Skepticism about cross-kingdom regulation is also discussed. In addition, the differences in miRNA biogenesis between plants and animals are analyzed, and the possible transport mechanisms between insects and plants are also discussed. Finally, the potential impact of utilizing miRNAs in agriculture is explored. The current review may provide a more profound understanding of the functions of plant-derived miRNAs in insects and enrich the knowledge of the growth and development regulation mechanisms of insects.

**Table 1:** Overview of the current status of cross-kingdom regulation of animal systems, especially insect systems, by plant-derived miRNAs.

miRNA	Host Plant	Target Organisms and Function	Reference
miR172	*Brassica oleracea*	The stomach, intestine, serum feces, blood, spleen, liver, and kidney of mouse	[13]
miR168a	Rice	Human/mouse low-density lipoprotein receptor adapter protein 1	[17]
miR159	*Arabidopsis thaliana* *Glycine max*	Human TCF7 that encodes a Wnt signaling transcription factor	[18]
miR5338	Rape	Mfn1 in the mouse prostate	[19]
miR7267-3p	Ginger	The *Lactobacillus rhamnosus* monooxygenase ycnE of human/mouse	[24]
miR396a-5p	Ginger	Inhibition of expression of Nsp12 in mouse	[25]
miR206	Sedr	Involved in hippo signaling pathway-fly, Wnt signaling pathway, and N-Glycan biosynthesis of honeybee	[26]
miR159a	*Arabidopsis thaliana*	Target the basic juvenile hormone-suppressible protein 1 gene of *Plutella xylostella*	[27]
agomir-7703-5p	*Arabidopsis thaliana*	Inhibition of the expression of phenoloxidase subunit 2 gene of *Plutella xylostella*	[27]
sbi-miR1-3p, sbi-miR2-3p, sbi-miR3-5p, sbi-miR5-5p, sbi-miR166-3p, sbi-miR390-5p, sbi-miR396-5p, sbi-miR2927-5p, sbi-miR6230-3p, sbi-miR6230-3p, sbi-5163-3p, hvu-miR3-3p, hvu-miR2-5p	Sorghum or barley	involved in detoxification of *Schizaphis graminum* and *Sipha flava*, such as metabolism of xenobiotics by P450s	[28]
miR162a	Cole *(Brassica campestris)* flower	Suppressing endogenous mTOR expression of *Apis mellifera* and *Drosophila*	[29]
Csu-novel-260	Insect-resistant genetically engineered rice	Suppressing the expression of the disembodied gene expression of *Chilo suppressalis*	[30]

## 2. Comparison of miRNA Biogenesis and Mechanisms in Plants and Insects

**Figure 1:**
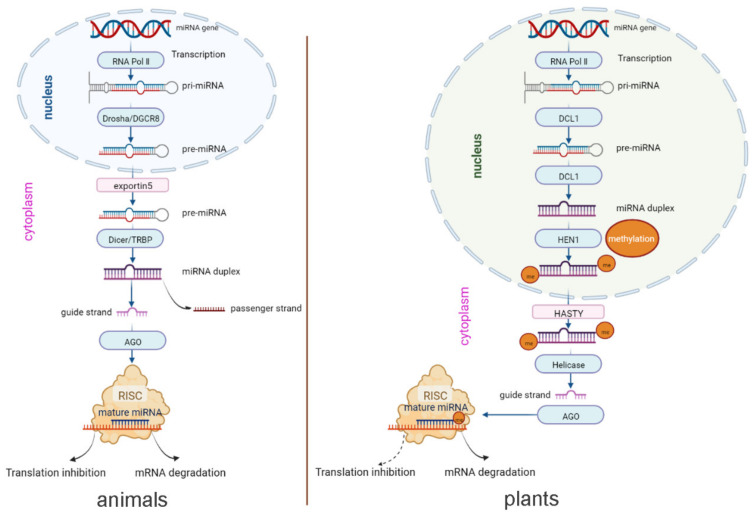
miRNA biogenesis pathways in animals and plants (the figure was drawn based on Samad et al. and Krol et al. [32,33]).

The biogenesis and other mechanisms of animal- and plant-derived miRNAs are similar overall. The process of biogenesis begins in the nucleus. As shown in Figure 1, in both animals and plants, capped and polyadenylated primary miRNA (pri-miRNA) transcripts are first transcribed by miRNA-encoding genes with the help of RNA polymerase Ⅱ, followed by removal of the hairpin stem to form the precursor-miRNA (pre-miRNA) and then further cleavage of the hairpin loop, resulting in the miRNA duplex [23]. Mature guide miRNAs are loaded on Argonaute (Ago) proteins to form the RNA-induced silencing complex (RISC) that binds to mRNA via target sequence complementarity to repress gene expression through inhibition of translation or degradation of mRNA [31].

However, the biogenesis of animal and plant-derived miRNAs biogenesis exhibits some distinct features. In plants, pri-miRNAs are cleaved through the action of the endonuclease DCL1 until they become mature miRNA duplexes. Subsequently, methylation at the 3′-terminus of the duplex is carried out by HEN1. Then, HASTY exports the methylated miRNA duplex from the nucleus to the cytoplasm [32]. In contrast, in animals, pre-miRNAs are produced through the activity of endonuclease Drosha and exported from the nucleus to the cytoplasm by the transport protein exportin5. Then, endonuclease Dicer converts the pre-miRNAs into mature miRNA duplexes [33]. To sum up, first, the maturation of miRNA occurs in the nucleus in animals but in the cytoplasm in plants. Second, the types of enzymes are different in animals and plants, although their functions are similar. In addition, in plants, the dominant mechanism (solid arrow) occurs via mRNA cleavage, in which the miRNA binds its target with high complementarity, while the alternative mechanism (dashed arrow) occurs by translational inhibition (Figure 1) [32].

## 3. Coevolution between Insects and Plants

Coevolution is the process in which two or more distinct taxa radiate and speciate in association with one another. The huge number of species of plants and insects is thought to be the result of adaptive radiation driven by the coevolution between plants and their beneficial animal pollinators or foragers [34]. In 1964, Ehrlich and Raven published “*Butterflies and Plants: A Study in Coevolution*” [35]. In this paper, the authors fostered a new way of thinking about the ecology and evolution of plant–herbivore interactions. Ehrlich and Raven theorized on the importance of coevolution to both the origin of species and the maintenance of species diversity, and suggested that coevolution between plants and their insect herbivores could drive the adaptive diversification of both groups. This theory laid the foundation for over five decades of research on anti-herbivore defense and coevolution.

In the course of developing and refining coevolutionary theory, plant secondary metabolites are considered defense compounds because they deter herbivory, reduce food digestibility, or directly interact with molecular targets in non-adapted insects [36]. In order to survive, insects must evolve to resist the plant’s secondary metabolites. The never-ending arms race drives coevolution between pathogens and hosts. In other words, insect herbivores can drive real-time ecological and evolutionary change in plant populations. In 2016, Marquis et al. [37] coupled the evolution of novel plant chemistry with reproductive isolation, thereby enabling plant speciation. According to this theory, the evolution of plant defenses could be associated with plant speciation. Meanwhile, phytophagous insects have been adapting to exploit their hosts [38]. Insects have developed sophisticated morphological, behavioral, and physiological adaptations that enable them to exploit plants as a resource [36]. The model system of Heliconiines and *Passiflora* (*Passiflora quadrangularis* L., 1758) (Magnoliatae: Malpighiales) plants was one of the first examples used to exemplify coevolution theory. In this model system, *Passiflora* plants evolved yellow structures mimicking heliconiine eggs and their extensive diversity of defense compounds known as cyanogenic glucosides. However, after a complex process of coevolution, Heliconiines can synthesize cyanogenic glucosides themselves, and the *Heliconius* (Insecta: Lepidoptera) adults have highly accurate visual and chemosensory systems. Further, the expansion of brain structures that can process such information allows *Heliconius* adults to memorize shapes and display elaborate pre-oviposition behavior [39]. Under some circumstances, plant defenses may impact pollinator health, foraging behavior, and reproductive success. Therefore, in order to survive and reproduce, flowering plants must balance the conflicting selective pressures of herbivore avoidance and pollinator attraction [40]. In brief, plants and animals have always been in a dynamic process of coevolution.

## 4. The Coevolution of miRNAs and miRNA Targets

Because plants are hosts for many species of insects, the coevolution between plants and insects is a complementary process, involving molecular pathways comprising important interactions between plants and insects. The non-coding RNAs, miRNAs, serve as a paradigm for studying functional divergence between paralogs and the possible coevolutionary processes between the duplicated miRNAs and the genomic contexts [41]. Studies of cross-kingdom regulation suggest that miRNAs play a significant role in the process of coevolution. The changes in the expression patterns of an existing miRNA may affect new sets of targets, forming novel regulatory circuits and changing pre-existing ones [42]. In other words, to maintain their regulatory functions, miRNAs have been forced to coevolve with their target genes when the targets experience functional divergence [43], which may be the molecular mechanism through which coevolution occurs.

The coevolution events for specific miRNAs and their targets in *Drosophila* and for miRNA-941 in primate evolution have been predicted and validated, although predictions of coevolution were limited to a few miRNAs and their target sites [44]. Barbash et al. [42] identified the coevolution of miRNAs and their targets by performing detailed genomic dissection using a combination of computational approaches. In addition, the evolutionary patterns of miRNAs and their targets during soybean domestication have been revealed through a comprehensive investigation of their genetic diversity [45]. Barik et al. [46] found that conserved miR167 sequences and their target auxin response factors (ARFs) have undergone coevolution, leading to functional diversification among diverse plant species. A family of X-linked miRNAs named spermatogenesis-related miRNAs also underwent strong coevolution with their target genes [47]. In a fungus–plant model (*Botrytis cinerea* and *Arabidopsis thaliana*) of RNA-based communication, the majority of sequences that were predicted to target host genes were shown to come from retrotransposons and intergenic regions in the fungal genome [48].

However, to date, no study has demonstrated that miRNAs play an important role in the coevolution of animals and plants. The cross-kingdom regulatory effects of plant-derived miRNAs on insects are, however, undisputed. Claycomb [49] postulated that extracellular small RNAs act as part of a pathogen’s arsenal by binding to target mRNA, thus enabling coevolution. Most studies emphasize the interaction between plant secondary metabolites and insect stress resistance. For example, in protein-related studies, the coevolution between viruses and hosts has been proven. The sequence of interactions that occur between MICA, a key natural killer (NK) cell activating receptor that recognizes a family of stress-induced ligands, and human cytomegalovirus, illustrates the dynamic and ongoing coevolution of virus and host, which enables the former to be so exquisitely tailored to the latter [50]. As knowledge in the field develops in depth, the cross-kingdom functions of miRNAs in coevolution will be further revealed. In the following sections, we summarize and discuss the cross-kingdom regulatory roles of plant miRNAs in insects to provide new insights into their coevolution.

## 5. Regulation of Insect Development by Plant-Derived miRNAs

Growing evidence has revealed that miRNAs target not only endogenous genes, but also exogenous genes. The cross-kingdom regulation capacity of plant-derived miRNAs has also been demonstrated [17]. There have been many studies about the effects of cross-kingdom regulation of plant-derived miRNAs on animal gut microbiota and diseases, including cancer and even COVID-19 [24,25,51]. In fact, it has been shown that only mature miRNAs from plants have cross-kingdom regulatory abilities; pre-miRNA, double-stranded miRNA, single-stranded miRNA, and DNA precursor miRNA do not have these abilities [17]. The cross-kingdom regulatory function of plant-derived miRNAs is attributable to their high stability, and these exogenous compounds can survive in the gastrointestinal tracts of animals [13]. Since the discovery of insect miRNAs in *Drosophila melanogaster*, miRNAs have been found to play an essential role in insect metamorphosis and reproduction [52,53]. In foliar or root-feeding insects, the release of cellular contents during tissue ingestion may trigger the delivery of plant-derived miRNAs. In phloem feeders, miRNAs must be present in the viscous sap and ingested by insects [23].

In recent years, a growing body of research has revealed the interaction between plants and insects. Soaking rice and maize root in dsRNA-containing solution leads to dsRNA absorption and increased insect mortality, which indicates that dsRNA could be transferred in the environment–plant–animal chain [54]. Gharehdaghi et al. [26] detected at least 11 plant miRNAs in the midgut of honey bees (*Apis mellifera* Linnaeus, 1758) (Insecta: Hymenoptera) fed sunflower and sedr pollen, and some target genes of these miRNAs are significantly involved in the hippo signaling pathway fly, Wnt signaling pathway, and N-glycan biosynthesis. In another study, plant-derived miRNAs were identified in the hemolymph of a cruciferous specialist *Plutella xylostella* (Insecta: Lepidoptera), indicating that these miRNAs could enter the circulatory system by penetrating the midgut barrier, and potentially play a regulatory role in *Plutella xylostella* [27]. In two cereal aphids, *Schizaphis graminum* (Insecta: Homoptera) and *Sipha flava* (Insecta: Homoptera), 13 sorghum miRNAs and three barley miRNAs have been detected and are predicted to target aphid genes involved in detoxification, and starch and sucrose metabolism [28]. It is known that in bee colonies, queens and workers develop from fertilized eggs and are thus genetically identical, but differ in terms of their morphology, physiology, and social function. Zhu et al. [29] found that miR162a in larval food could regulate honey bee caste development by targeting the mTOR gene and suppressing endogenous *dmTOR* expression in non-social *Drosophila melanogaster* (Insecta: Pterygota). MiR162a also causes extended developmental times and reductions in body weight and length, ovary size, and fecundity in *Drosophila* larvae. This study found that honey bee caste development could be regulated by plant-derived miRNAs, offering insights for understanding cross-kingdom interactions. Recent research has also found that the plant miRNA response and miRNA-mediated post-transcriptional regulation during phloem-feeding insect infestation are similar to those during pathogen invasion [55]. In addition, overexpression of insect endogenous miRNA in transgenic rice inhibits the pupation of *Chilo suppressalis* (Insecta: Pterygota) and *Cnaphalocrocis medinalis* (Insecta: Lepidoptera), which also proves that cross-kingdom regulation of miRNAs from plants could be carried out [30].

The engineering of plants to express dsRNAs targeting vital insect genes in order to confer protection from herbivorous insects is still challenging. The low level of intact dsRNA in plant-derived foods does not trigger potent RNAi in the pest [56]. To date, as shown above, some studies have identified plant-derived miRNAs in insects; putative targets also have been bioinformatically predicted, and a few studies have proven that plant-derived miRNAs can regulate insect development. The studies of cross-kingdom regulation between plant-derived miRNAs and insects have not only enriched the knowledge of cross-kingdom regulation, but also laid the foundation for understanding the coevolution and information exchange mechanisms between insects and plants. In the food chain, insects, especially herbivorous insects, are associated with plants. Over a long period of coevolution, the interaction between plant-derived miRNAs and insects may become more complex, and further studies will reveal the cross-kingdom regulation mechanisms.

## 6. Insect-Derived miRNAs Targeting Plant Defense Response

When insects feed on plants, insect-derived miRNAs inevitably enter the plant through saliva. Therefore, information exchange between plants and insects is inevitable during this process. In addition, pathogens, parasites, and viruses act as vectors for miRNA transmission between plants and insects. Some plant pathogens and pests are capable of delivering miRNAs into host cells to modulate the host immune response [57,58]. Over the course of long-term evolution, plants have established appropriate self-defense mechanisms to defend themselves against insect attacks [59]. MiRNAs produced by plants to interfere with insect herbivores need to be able to access insect tissues. As mentioned in regard to coevolutionary relationships between insects and plants, insect oral secretions contain specific chemicals that are likely to have evolved as effectors to inhibit plant defenses and, over time, some plants have adapted to recognize some of these substances so that they may even trigger defense responses [60]. Insects have also evolved related strategies to detoxify plant-specialized metabolites [61]. Insects mitigate the potentially dangerous effects of these compounds by either moving to a different plant source or detoxifying the compounds with enzymes such as cytochrome P450s (CYP), esterases, or glutathione S-transferases (GST) [23]. These detoxification enzymes may be regulated by endogenous miRNAs, particularly the CYPs, as part of the insect offenses to plant defenses. For example, effectors in caterpillar regurgitant could modify plant responses to herbivory by influencing defense signaling through phytohormone crosstalk [62] in either negative or positive manners. In the tobacco-silenced RdR1 (irdr1) lines, artificial damage and treatment with caterpillar regurgitant resulted in lower jasmonate and higher ethylene levels at 30 and 300 min after wounding, respectively, compared to the wild type, implying that miRNAs in the caterpillar regurgitant could influence the expression of plant miRNAs [63]. Recently, the miRNA profiles of *Helicoverpa armigera* (Insecta: Lepidoptera)-infested leaves and adjacent undamaged leaves of *Cajanus scarabaeoides* (Magnoliatae: Fabales) were compared to identify dynamic miRNA molecules that potentially act as mediators of systemic defense responses. The results showed that most of the miRNA detected in the adjacent leaves targeted genes involved in the defense pathways and plant immune response [64], indicating that insect-derived miRNAs could trigger defense responses in plants.

Studies exploring the targeting of the plant defense response by insect-derived miRNAs are limited. Perhaps because there are so many insect species in contact with each plant, the effect of insect-derived miRNAs on plants has been neglected. However, the spread of some plant diseases is involved in the activity of insects or the pathogens and parasites of insects. While focusing on plant-derived miRNAs’ regulation of insect development, the effects of insect-derived miRNAs on plants cannot be ignored.

## 7. The Cross-Kingdom Transport Mechanism of Plant-Derived miRNAs

As mentioned above, the environment of the digestive system in insects is complex. Therefore, if plant-derived miRNAs are taken up and eventually reach the target cell, they will overcome numerous challenges, including digestive enzymes and the intestinal wall barrier. The possible mechanisms involved in miRNA transport may be transmembrane channel-mediated mechanisms based on RNA transporter-like proteins or microvesicle-mediated [16,23].

SID-1 (systemic RNAi deficient-1), a multipass transmembrane protein, is believed to form a channel that could allow passive diffusion of dsRNA in mammalian cells and *Caenorhabditis elegans* [65]. SID-2 is another recently identified transmembrane protein in *C. elegans* [66]. Microvesicles (MVs), membrane-covered vesicles that can be released by various kinds of cells [67], can be divided into shedding vesicles, exosomes, and apoptotic bodies based on their mechanism of formation and their intracellular origin [68]. The miRNAs are packed in MVs, which can help miRNAs escape RNase digestion [69], indicating that the MV structure is critical for miRNA stability.

**Figure 2:**
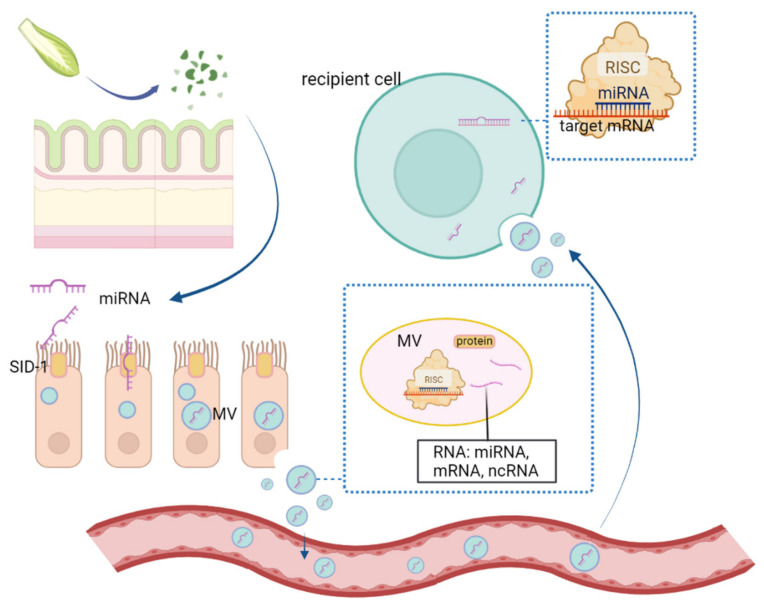
Potential mechanisms by which plant-derived miRNAs regulate insect target genes (the figure was drawn and modified based on Chen et al. [16]).

As shown in Figure 2, one possible mechanism may be that the intestinal epithelial cells can take up plant miRNAs from food. After feeding, the dietary miRNAs are released into the digestive tract by mechanical digestion. In such an environment with an unsuitable pH, mature miRNAs escape from degradation by nuclease and are efficiently absorbed via SID-1 and SID-2 proteins located in the plasma membrane. The miRNAs are selectively packaged into MVs, which are secreted from pit cells, together with components of the RISC. Then, the secreted MVs undergo exocytosis from pit cells, and then are pooled into the circulatory system and delivered to other tissues and organs. On reaching the final recipient cells through endocytosis, miRNAs are released and regulate the target gene in a sequence-specific manner [16].

## 8. Controversy Regarding the Modulation of Insect Development by Plant-Derived miRNAs

Any theory must undergo a long period of development and maturity. The concept of cross-kingdom regulation has been controversial since the first identification of the cross-kingdom regulation of plant-derived miRNAs [17] because some groups of researchers found that these results lack reproducibility. In a study, although plant-derived miR168 was over-represented in public sRNA datasets from various tissues of mammals, chickens, and insects, miR168a was also detected in insects that were not fed monocot plants. Researchers thought that plant miRNAs in animal sRNA datasets may originate during the process of sequencing [20]. Analysis of coleopteran insects sensitive to environmental RNAi fed on wild-type plants revealed the uptake of plant endogenous long dsRNAs, but not small RNAs [70]. In addition, a study found that caterpillars remain recalcitrant to the effects of either longer dsRNA or miRNA, indicating the harsher environment of the gut where the advanced peritrophic membrane may limit miRNA availability [71]. Additionally, the alkaline environment of the gut also hampers the stable existence of miRNAs. Some research has reported that the systemic uptake of foreign miRNAs from the digestive tract is negligible and significantly below the levels that are required to be biologically relevant when acting through canonical sequence-specific miRNA-mediated mechanisms [72].

Chen et al. [73] demonstrated that a microRNA-mediated RNAi-based insect-resistant genetically engineered rice line expressing endogenous *Chilo suppressalis* Csu-novel-260 shows significant resistance to target pests. However, *Apis mellifera* adults are not susceptible to high doses of Csu-novel-260, and the impact of miRNA-mediated RNAi-based insect-resistant genetically engineered plants on *Apis mellifera* is negligible. In honey bees, after oral uptake of pollen containing miR156a, miR159a, and miR169a, negligible delivery of these molecules is observed in recipient honey bees [72]. There was no evidence of delivery of biologically relevant miRNAs in proximal or distal tissues of recipient honey bees [74]. Researchers also found that although several mulberry-derived miRNAs could enter silkworm hemolymph and multiple tested tissues, these miRNAs did not play roles in silkworm physiological processes [75].

Considering these controversies together, there are some important points to explore [51]. The first concerns the construction and sequencing of plant sRNA libraries. Plant miRNA libraries are not constructed with the same precision due to the presence of 2′-O-methylated 3′ end modification [76]. Another is that nucleic acids are common contaminants in experiments involving genomic library preparation and sequencing, thereby producing false positive results [77]. In addition, there is, admittedly, little research to prove that plant-derived miRNAs regulate insect development. Although our understanding of cross-kingdom gene regulation remains inconclusive [78], increased research and advances in sequencing technology should resolve these controversies.

## 9. Conclusions and Future Prospects

In conclusion, the discovery of cross-kingdom regulation of gene expression by plant-derived miRNAs has generated considerable excitement. Plants and insects take advantage of miRNA regulation of physiological processes to manipulate each other, and the patterns of cross-kingdom regulation have important significance for pest control. Transgenic corn plants engineered to express western corn rootworm *Diabrotica virgifera virgifera* Leonte (Insecta: Coleoptera) dsRNAs show a significant reduction in feeding damage, indicating that the RNAi pathway could be exploited to control insect pests via *in planta* expression of a dsRNA. Further, dsRNA spraying is an effective method for pest control [79,80,81]. Clarifying the cross-kingdom regulation mechanism will not only help clarify the coevolutionary history of plants and animals, but also illuminate the possible pathways by which miRNA regulates the growth and development of insects. However, there are some controversies regarding the modulation of insect development by plant-derived miRNAs, and more studies are needed to explore the mechanisms in deeper detail. The variety and abundance of insects and plants provide models for research. There is little doubt that miRNA has huge potential for application to influence biological functions in the host through mediating cross-kingdom regulation by integrating into a specific target gene-mediated regulatory pathway or a complex regulatory network. Cross-kingdom regulation of key physiological processes by miRNAs makes dsRNAi applications an attractive target. The natural uptake and utilization of miRNAs in plants by herbivorous insects can become a novel avenue to understand complex plant–insect interactions. In the future, a greater understanding of miRNA-mediated cross-kingdom regulation will result in the application of miRNAs for dietary therapy, pest management, and promoting pollination by insects, further enriching coevolution theory.

## Data Availability

Data sharing not applicable.
